# Treatment with omega-3-rich fish oil enhances host defense and reduces intestinal colonization by diarrheagenic *Escherichia coli*

**DOI:** 10.1590/1414-431X2025e14855

**Published:** 2025-10-17

**Authors:** T.A.V. Câmara, B.G. Vila Nova, M.C.C. Costa, A.R.S. Ares, M. Andrade-Silva, I.S.C. da Silva, R.G. Assunção, J.C.S. Sousa, A.G. Abreu

**Affiliations:** 1 Programa de Pós-Graduação em Ciências da Saúde, Universidade Federal do Maranhão, São Luís, MA, Brasil; 2 Laboratório de Imunofisiologia, Universidade Federal do Maranhão, São Luís, MA, Brasil; 3 Laboratório de Patogenicidade Microbiana, Universidade Ceuma, São Luís, MA, Brasil; 4 Departamento de Morfologia, Universidade Federal do Maranhão, São Luís, MA, Brasil

**Keywords:** Intestinal integrity, Enteroaggregative *Escherichia coli*, Polyunsaturated fatty acids, Omega-3

## Abstract

*Escherichia coli* is a common intestinal microorganism that can cause a variety of diseases in humans and animals. The aim of this study was to evaluate the therapeutic effects of fish oil rich in omega-3 against intestinal infection caused by enteroaggregative *E. coli* (EAEC). Minimum inhibitory and bactericidal concentrations were determined, along with toxicity assays using HT-29 intestinal cells and *Tenebrio molitor* larvae. Swiss mice infected with EAEC 042 were used to assess the *in vivo* therapeutic potential of fish oil. Histological analyses of the liver, kidney, and colon were conducted to identify tissue alterations such as inflammation and necrosis. Fish oil exhibited a bacteriostatic effect on *E. coli* and was non-toxic to HT-29 cells at concentrations up to 50 mg/mL. It also enhanced survival in treated larvae. In infected mice, bacterial colony counts were significantly lower in the fish oil-treated group. Histological evaluation showed reduced inflammatory infiltrates in the liver and colon, and no progression of hepatic hydropic degeneration was observed in treated animals, unlike in the untreated infected group. These findings indicated that fish oil rich in omega-3 possesses antimicrobial activity against *E. coli,* is non-toxic to both cells and animal models, and effectively reduces intestinal infection and associated tissue damage in mice. This suggests its potential as a supportive therapeutic agent for infections caused by pathogenic *E. coli*.

## Introduction

Diarrheal diseases remain a significant cause of morbidity and mortality worldwide, particularly affecting hospitalized patients and populations in underdeveloped and developing regions (1). Among the most vulnerable people are children under five years of age, for whom diarrhea is the second leading cause of death, surpassed only by pneumonia, making it a major public health concern (2).

Clinically, diarrhea is characterized by an increased frequency of watery stools, often accompanied by fever, vomiting, and abdominal pain. Although most cases are self-limiting, resolving within 2 to 14 days, a subset can persist beyond this period (3). Persistent diarrhea is defined as the presence of three or more loose or liquid stools per day for more than two weeks (4). In these cases, the condition is usually of infectious origin and may lead to chronic enteropathy, where the mucosal damage impairs the digestive and absorptive functions of the gastrointestinal tract (5).

Among the primary bacterial agents implicated in such conditions are enteroaggregative *E. coli* (EAEC) and enteropathogenic *E. coli* (EPEC). Poor nutritional status is recognized as the most significant epidemiological risk factor for the development and progression of persistent diarrhea (6). *E. coli* can disrupt the epithelial barrier, promoting bacterial persistence, invasion, and tissue dissemination (7). Moreover, infections caused by diarrheagenic *E. coli* place considerable demand on outpatient services and healthcare systems, leading to substantial economic burdens (1).

Intestinal health depends not only on a balanced microbiota but also on the structural integrity of epithelial cells, both of which are influenced by diet (8). The quality and quantity of specific nutrients can create environments that either support or hinder microbial growth and epithelial integrity (9).

Recent research has highlighted the role of dietary lipids in modulating the gut microbiota and maintaining the architecture of the intestinal barrier (10). Polyunsaturated fatty acids (PUFAs), particularly omega-3 (n-3) types, are key components of cellular phospholipids. They are critical for membrane fluidity and cell signaling and serve as precursors for bioactive lipid mediators, contributing directly to epithelial cell function and protection (11,12).

Given the ability of n-3 PUFAs to promote intestinal cellular integrity and inhibit the proliferation of pathogenic bacteria, the present study aimed to evaluate the therapeutic effect of fish oil rich in omega-3 fatty acids on intestinal infection caused by EAEC.

## Material and Methods

### Microorganism

In this study, the EAEC 042 strain was used to evaluate the effect of fish oil rich in omega-3. The strain was kept at -80°C in trypticase soy broth (TSB) supplemented with 20% glycerol. For experimental procedures, the strain was cultured in Luria-Bertani (LB) broth, LB or MacConkey agar plates, supplemented with appropriate antibiotics when necessary.

### Fish oil

A commercial fish oil formulation (FDC^®^, Biowell America LTDA, Natal, Brazil), available in 1000-mg capsules, was used. Its composition consisted of 13.6% docosahexaenoic acid (DHA), 18.6% eicosapentaenoic acid (EPA), and 68.3% total omega-3 fatty acids. For the *in vivo* experiments, fish oil was administered orogastrically via gavage to mice.

### Minimum inhibitory concentration (MIC) and minimum bactericidal concentration (MBC)

The MIC of fish oil was determined by standard broth microdilution assays. EAEC 042 was grown overnight (18-24 h) at 37°C in Mueller Hinton (MH) broth. The bacterial culture was then diluted and grown to mid-log phase, reaching an absorbance (OD_600_) of approximately 0.08-0.13, corresponding to ∼1.5×10^8^ colony forming units (CFU)/mL. Serial dilutions of fish oil (ranging from 0.04 to 100 mg/mL) were prepared in MH broth (100 μL per well), followed by the addition of 100 μL of each dilution into a 96-well plate. Subsequently, 10 μL of the bacterial inoculum was added to each well. The plates were incubated at 37°C for 16-20 h. Following incubation, 50 μL of 0.03% resazurin solution was added to each well and incubated for 1 h. The MIC was defined as the lowest concentration of the fish oil that prevented a color change from blue to pink, indicating inhibition of bacterial metabolic activity (13).

For MBC determination, 10 μL aliquots from wells with no visible growth were plated onto MH agar and incubated at 37°C for 18-24 h. The MBC was defined as the lowest concentration at which no colony formation was observed. All the experiments were performed in triplicate and repeated at least twice to ensure reproducibility.

### Cytotoxicity

HT-29 cells were used to evaluate the cellular cytotoxicity. Cells were stored in liquid nitrogen and were aliquoted in 1 mL of appropriate freezing medium (95% fetal bovine serum + 5% dimethyl sulfoxide - DMSO) at a proportion of 1×10^6^ cells per tube. For cell cultivation, Dulbecco modification of minimum essential media (DMEM) was used with 10% fetal bovine serum and 1% antibiotic (streptomycin sulfate and penicillin). Approximately 1 mL of HT-29 cells (frozen at -80°C) with 5 mL of DMEM were placed in the culture bottle to observe cell growth. After an incubation period of two days, it was possible to observe a homogeneous monolayer.

Cells were treated with fish oil at concentrations of 50, 25, 12.5, 6.25, 3.12, 1.56, 0.78, 0.39, 0.19, and 0.09 mg/mL and incubated for 24 h. After the incubation period, 100 µL of MTT (3-(4,5-dimethylthiazol-2-yl)-2,5-diphenyltetrazolium bromide) was added to each well, and the plates were incubated for 3 h at 37°C in the oven. Then, 100 µL of DMSO was added to each well, and the samples were homogenized to completely dissolve the formazan crystals. The content of each well was subjected to absorbance determination at 550 nm (14).

The results were calculated from the absorbance measurement using the formula [1 - (Abs exp. / Abs contr.)] × 100, with the percentage of the absorbance measurement of the cells treated with the compounds in relation to control cells (without treatment). Experiments were performed in triplicate.

### Survival assay in *Tenebrio molitor* larvae

Toxicity analysis was performed with *T. molitor* larvae. Larvae weighing approximately 100 mg were placed in properly identified petri dishes and randomized into 6 groups, each containing 10 units: phosphate buffered saline (PBS)_1_: single puncture control; PBS_2_: double puncture control; *E. coli*: infected with EAEC 042; Fish oil: fish oil only; Treatment: *E. coli* followed by fish oil (2-h interval); Prophylactic: fish oil followed by *E. coli* (2-h interval).

To administer the treatments, insulin syringes (6×0.25 mm) were used to inject 10 µL of the prepared solutions (fish oil, PBS, or bacterial suspension). A suspension of *E. coli* strain EAEC 042 was prepared with an absorbance of 0.7 at 600 nm and used for infection in the appropriate groups. A 2-h interval was maintained between the administration of fish oil and bacterial infection for both prophylactic and therapeutic protocols. In cases of a single perforation, injections were made into the last abdominal ring of the larvae; dual perforations were made in the last and penultimate rings. Negative control groups received PBS with identical puncture patterns. Following treatment, all larvae were maintained at room temperature for 10 days. Survival was monitored daily, with non-responsive or dead larvae counted every 24 h to assesses survival rates (15).

### Intestinal colonization of mice infected with *E. coli* 042

All experimental procedures involving animals were approved by the Ethics Committee on Animal Experimentation of CEUMA University (Protocol No. 75/19) and conduced in accordance with the ethical and technical guidelines established by the Brazilian Society of Laboratory Animals Science.

Female Swiss mice (8-12 weeks old, ∼25 g) were obtained from the Central Animal House of the CEUMA University (São Luís, Brazil). Animals were maintained under controlled conditions: 26±2°C, 44-56% relative humidity, with a 12-h light/dark cycle. Sterile food and water were provided *ad libitum*.

The streptomycin-treated mouse model adapted from Harrington et al. (16) was used to evaluate the intestinal colonization by EAEC 042. Mice received drinking water supplemented with streptomycin (5 g/L) for 48 h before bacterial inoculation and continued on the same treatment for the duration of the experiment. This selective pressure was used to enhance colonization by EAEC 042, which is resistant to streptomycin.

The animals were randomly divided into 4 groups (n=6): PBS; Fish oil; *E. coli* 042; and EAEC 042 + fish oil (infected and treated daily with fish oil).

Prior to infection, food was suspended for 1:30 h, and the mice were orally administered 200 µL of a 0.4 M sodium bicarbonate solution to reduce gastric acidity. After 15 min, 200 μL of EAEC 042 inoculum (5×10^3^ CFU/mL) was administered via oral gavage.

Fresh fecal samples were collected daily for 15 days post-infection. Samples were weighed, diluted, and homogenized in sterile PBS. Serial dilutions were plated on MacConkey agar supplemented with streptomycin (100 mg/mL) to determine CFU/mL. Colonization by EAEC 042 was confirmed by PCR targeting the *pic* gene, a known virulence marker for this strain.

### Histological analysis

The collected samples - kidney, liver, and intestine (colon) - were fixed in a 10% formaldehyde solution for 24 h. They were sectioned and processed for inclusion in paraffin. Sections of 5 mm were stained with hematoxylin-eosin for histopathological analysis. The following parameters were evaluated: edema, necrosis, cellular infiltrate, and hemorrhage. The classification (0=absent; 1=slight; 2=moderate; 3=intense) was performed according to that described by Liberio et al. (17).

### Statistical analysis

All statistical analyses were conducted using GraphPad Prism software (USA), version 7.0. The Shapiro-Wilk test was employed to assess data normality. For the acute toxicity assay in *T. molitor* larvae, survival rates were analyzed using the log-rank (Mantel-Cox) test to compare survival curves between groups. Cell viability data were analyzed using one-way analysis of variance (ANOVA) followed by Tukey's *post hoc* test to determine significance between treated groups. For the quantification of CFU in fecal samples from mice, two-way ANOVA for repeated measures was applied, followed by Tukey's *post hoc* test for multiple comparisons. Results were considered statistically significant at P≤0.05.

## Results

### Fish oil rich in omega-3 inhibited bacterial growth

Fish oil, characterized by a high content of omega-3 fatty acids, demonstrated inhibitory activity against EAEC 042. Bacterial growth was suppressed at concentrations equal to or greater than 25.0 mg/mL. Regarding MBC, bacterial growth was inhibited only at a concentration of 100 mg/mL.

### Fish oil was not cytotoxic to HT-29 cells

**Figure 1:**
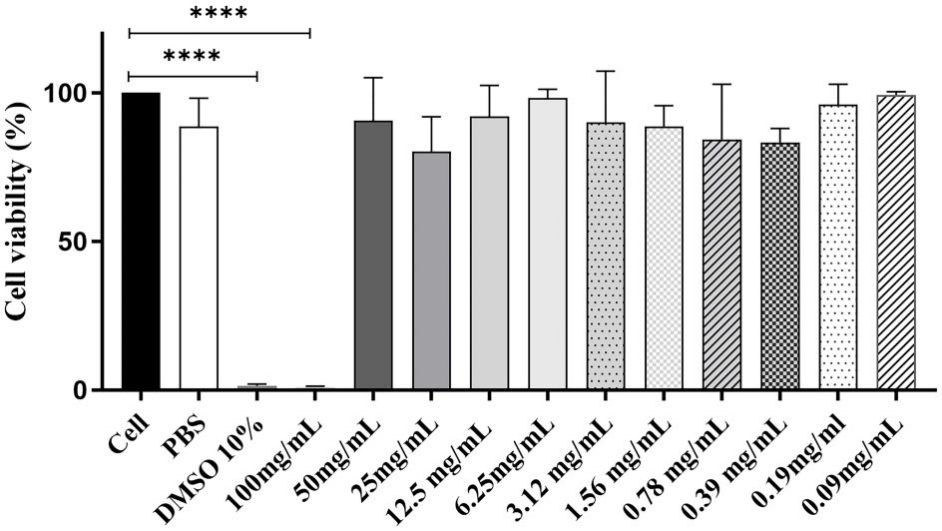
Viability of HT-29 cells at different fish oil concentrations. Data are reported as means and SD. ****P<0.001 compared to the HT-29 cell and phosphate buffered saline (PBS) groups (one-way ANOVA followed by Turkey's multiple comparison test).

To evaluate the cytotoxic potential of fish oil, an MTT assay was performed using HT-29 cells. As shown in Figure 1, no significant differences were observed for fish oil concentrations ranging from 0.09 to 50 mg/mL compared to control, indicating that the oil was non-toxic at these doses. However, the 100 mg/mL concentration exhibited cytotoxicity comparable to that of 10% DMSO.

### Fish oil increased the survival of *T. molitor* infected by *E. coli* 042

**Figure 2:**
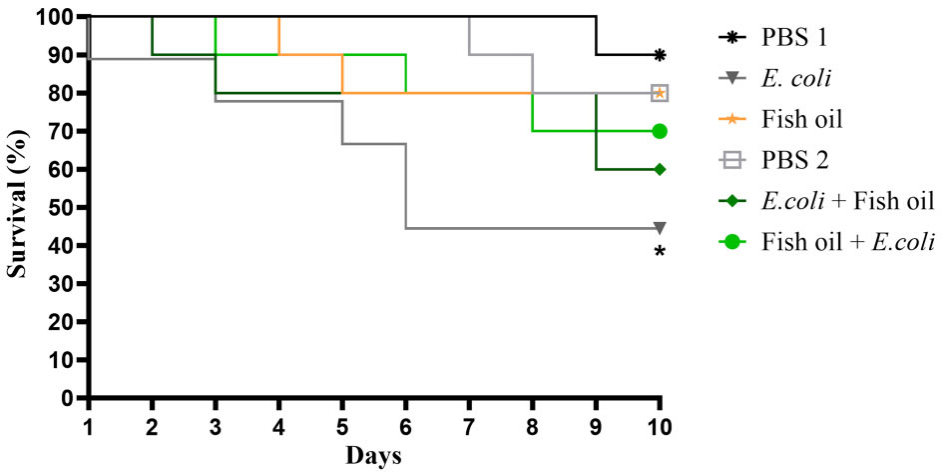
Survival rate (%) of *Tenebrio molitor* larvae over a 10-day observation period. *P<0.05 compared to the phosphate buffered saline (PBS) group. Group survival curves were compared using the Log-rank test (Mantel-Cox) test.

As observed in Figure 2, larvae infected with EAEC 042 showed a survival rate of 44% over the observation period. Treatment with fish oil, both as a post-infection therapy and as a prophylactic measure, resulted in a notable increase in larval survival. Importantly, fish oil alone did not induce toxicity, as evidenced by the similar survival rates between the fish oil group and the PBS group.

### Fish oil decreased intestinal colonization in mice infected with diarrheagenic *E. coli*

To evaluate the effect of fish oil on intestinal colonization by EAEC 042, fecal samples from infected mice were collected daily over a 15-day period. Colonization was confirmed by PCR amplification of the *pic* gene, a marker specific to the EAEC 042 strain.

**Figure 3:**
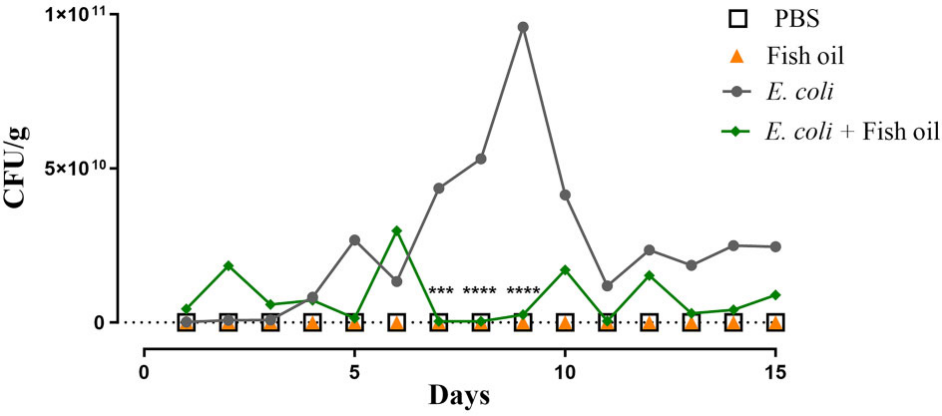
Intestinal colonization in fish oil-treated mice following infection with enteroaggregative *E. coli* (EAEC) 042. Data are reported as means±SD. ***P<0.001 and ****P<0.0001 compared to the untreated *E. coli* group (two-way ANOVA with Tukey's multiple comparison).

As shown in Figure 3, mice treated with fish oil exhibited significantly reduced CFU/g feces compared to the untreated infected group. Notably, statistically significant reductions in bacterial load were observed on days 7 (P<0.001), 8 (P<0.0001), and 9 (P<0.0001).

From day 9 onward, no detectable CFUs were observed in the fish oil-treated group, indicating a complete clearance of intestinal colonization. In contrast, the untreated infected group reached its peak on day 9 and only began to show a gradual decline thereafter. These findings suggest that fish oil rich in omega-3 exerts a protective effect against intestinal infection by *E. coli*.

### Fish oil treatment reduced inflammatory response in infected organs

Histopathological analysis revealed varying degrees of vascular congestion across all experimental groups, likely associated with the euthanasia process. Inflammatory infiltrate, predominantly composed of polymorphonuclear cells, was observed in the intestinal submucosal of both infected and non-infected animals.

In the group infected with EAEC 042, a moderate degree of intestinal inflammation was noted, along with hepatic inflammatory infiltrates - findings not present in the other groups. In contrast, animals infected with EAEC 042 and treated with fish oil showed only mild inflammatory infiltration in the liver, with no progression of hepatic hydropic degeneration. Additionally, one animal in this group exhibited only vascular congestion in the liver and kidneys, without other pathological alterations (Table 1).

**Table 1:** Histopathological evaluation of the liver, kidneys, and intestines of animals subjected to intestinal colonization by *E. coli* 042 and fish oil treatment.

	Congestion	Infiltrate	Degeneration
Liver			
PBS	1.0±0.0	0	0
Fish oil	1.0±0.0	0	0.3±0.6
*E. coli*	2.7±0.6^a^	0.7±0.6	1.3±1.1^e^
*E. coli* + Fish oil	1.3±0.6^b^	0.3±0.6	0.3±0.6
Kidneys			
PBS	1.0±0.0	0	0
Fish oil	1.0±0.0	0	0.3±0.6
*E. coli*	2.3±0.6^c^	0	0.7±0.6
*E. coli* + Fish oil	0.3±0.6^d^	0	0
Intestine			
PBS	0	0	0
Fish oil	0	0.3±0.5	0
*E. coli*	1.0±0.0	1.3±1.1^e^	0
*E. coli* + Fish oil	0	1.0±0.0	0

Data are reported as means±SD from five animals per group. The scoring system used was: 0=absent, 1=mild, 2=moderate, 3=intense. ^a^P<0.001 compared to the phosphate buffered saline (PBS) and Fish oil groups; ^b^P<0.05 compared to the *E. coli* group; ^c^P<0.05 compared to the PBS and Fish oil groups; ^d^P<0.001 compared to the *E. coli* group; ^e^P<0.05 compared to the PBS group. ANOVA.

**Figure 4:**
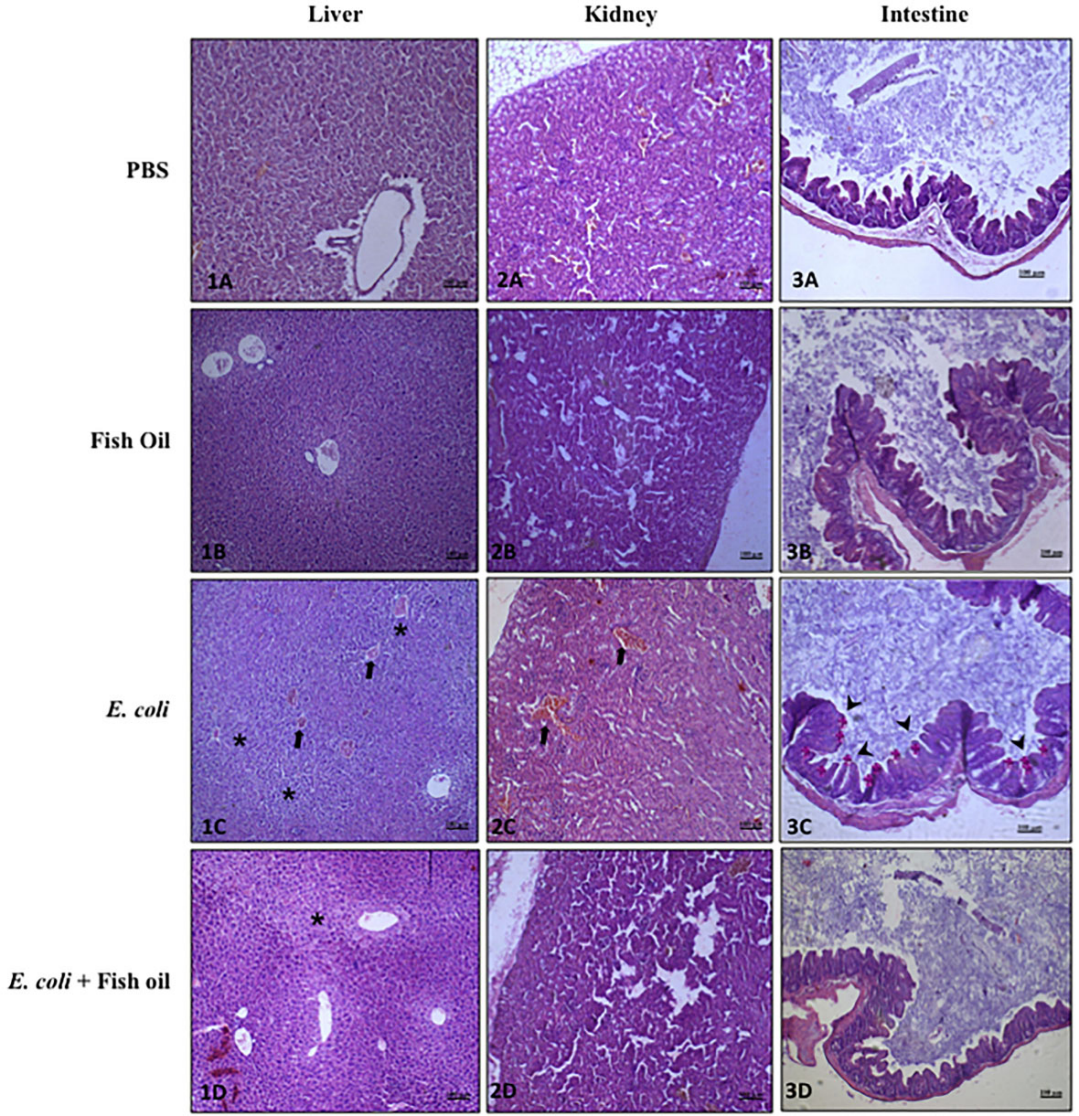
Histopathological analysis (HE) of liver, kidney, and intestinal tissues from animals infected with *E. coli* 042 and treated with fish oil. 1A, 2A, 3A: phosphate buffered saline (PBS); 1B, 2B, 3B: Fish oil; 1C, 2C, 3C: *E. coli* 042; and 1D, 2D, 3D: *E. coli* 042 + Fish oil. Indicators: congestion (arrow); hydropic degeneration (asterisk); inflammatory infiltrate in the intestinal submucosa (arrowhead). The scale bar is 100 µm.

Overall, no significant histological abnormalities were observed in the liver or intestinal tissue of the fish oil-treated group, suggesting a protective, anti-inflammatory effect of the treatment (Figure 4).

## Discussion

In this study, we investigated the biological activity and safety of fish oil rich in omega-3 fatty acids through a combination of *in vitro* and *in vivo* models. The results demonstrated that fish oil exhibited antimicrobial properties, reduced intestinal colonization by EAEC 042, and did not exert toxic effects on HT-29 cells or *T. molitor* larvae. These findings suggest the compound is a potential therapeutic or prophylactic agent against enteric infections.

When investigating the cytotoxicity on HT-29 cells, fish oil was found to be non-toxic at concentrations up to 50 mg/mL. These results are supported by Hao et al. (18), who investigated the protective effect of omega-3 fatty acid eicosapentaenoic acid (EPA) after adherent and invasive *E. coli* LF82 infection. After treatment with EPA at concentrations of 25 and 50 μg, cell proliferation was induced; however, the groups treated with EPA at concentrations of 100 and 200 μg showed a significant inhibitory effect on Caco-2 cell proliferation with an increased rate of cell apoptosis compared to the control group.

In the research by Xiao et al. (19), Caco-2 cells were pre-incubated with PUFAs abundant in fish oil, such as EPA and DHA. The results indicated that only the group treated with EPA showed a significant increase in the function of the intestinal epithelial barrier, which had been impaired by heat stress. In addition, EPA promoted a significant increase in the expression of cell junction proteins, occlusion, and ZO-1, while DHA demonstrated a lower efficacy in this aspect. Pretreatment with EPA also effectively prevented the degeneration of cell morphology, evidencing its protective role in stressed conditions.

In the *in vivo* toxicity evaluation using the alternative *T. molitor* larvae model, fish oil demonstrated a favorable safety profile. The survival rate of larvae treated with fish oil alone was comparable to that of the PBS control group, indicating no observable toxic effects. Furthermore, the antimicrobial potential of fish oil was evident, as the survival rate of larvae infected with EAEC 042 reached approximately 70% after either therapeutic or prophylactic treatment with the compound. In stark contrast, the group that received *E. coli* inoculation without any intervention exhibited the lowest survival rate. These results suggest that fish oil not only is non-toxic but also exerts a protective effect, potentially enhancing the host's resistance to pathogenic challenge.

The antimicrobial activity of fish oil against *E. coli* was found to be satisfactory, suggesting significant potential for both the prevention and treatment of infections. Das (20) proposes that this protective effect may stem from the generation of free radicals and lipid peroxides within the oil, which damage bacterial membranes. Furthermore, Clare et al. (21) demonstrated that key components of fish oil, such as DHA and EPA, exhibit potent inhibitory effects against *E. coli* and are also effective in eradicating bacterial biofilms.

While numerous studies have documented the anti-inflammatory and antimicrobial properties of fish oil, none have specifically evaluated its effects in the context of diarrhea diseases. Additionally, it is worth noting that no animal model to date fully replicates human diarrhea. However, the intestinal colonization model described by Harrington et al. (16) remains the most suitable protocol for assessing the persistence of pathogenic bacteria in the gastrointestinal tract.

EAEC is known to adhere to the host's intestinal epithelium, a critical step in colonization that contributes to various pathological changes (22). The virulence of EAEC is mediated by several factors that facilitate adhesion, internalization, and persistence, especially under conditions of gut microbiota imbalance and impaired immune system (23).

If the main physical barrier of the intestine (intestinal cells) is compromised, even minute amounts of endotoxins such as lipopolysaccharide (LPS) can reach systemic circulation and, even at a picogram level, have the potential to provoke a high inflammatory response (24). LPS enters the bloodstream through the intestinal epithelium or through the opening of tight junctions between epithelial cells. This highlights the importance of interventions that improve not only the profile and quantity of bacteria but also cell integrity and the control of local inflammation (22).

Studies indicate that fish oil supplementation can attenuate endotoxemia, which compromises the intestinal barrier, and improve immune function (25). Costanzo et al. (26) evaluated the effects of krill oil on intestinal inflammation, epithelial barrier integrity, and pathogenicity of adherent-invasive *E. coli* LF82. Their results showed that the oil reduced bacterial adhesion and invasion in epithelial cells and decreased intestinal inflammation. Krill is a crustacean that feeds on microscopic algae and produces significant amounts of omega-3 fatty acids. Due to its diet, krill accumulates EPA and DHA, making the oil extracted from this crustacean rich in these compounds. Thus, it represents an alternative source of EPA and DHA, similar to fish oil, promoting protection of the intestinal barrier (27).

In this study, animals colonized with EAEC 042 and treated with fish oil showed a significant reduction in bacterial colonization for several days compared to the untreated EAEC 042 group, demonstrating a protective effect on the intestinal tract of these animals. Another study showed that supplementation with krill oil, rich in omega-3 fatty acids, not only reduced bacterial adhesion and invasion but also inhibited the inflammatory response and preserved the integrity of the intestinal mucosa, promoting epithelial recovery (26).

The histopathological data from the present study revealed the presence of inflammatory infiltrates in the intestinal submucosa across all groups of animals. However, the infected group that did not receive fish oil treatment showed a more intense infiltration in the intestine compared to the other groups. Additionally, it was the only group to exhibit hepatic inflammatory infiltrates. These findings suggest a greater likelihood of increased intestinal permeability and, consequently, bacterial or toxin translocation, which could trigger systemic inflammatory responses.

Inflammation is a natural defense mechanism of the body against pathogenic organisms and other disruptions of homeostasis. It creates an environment hostile to pathogen survival, initiates immune defense responses, induces metabolic changes in the host, and promotes tissue repair, ultimately helping to restore balance in infected or injured areas (28). However, a more severe inflammatory process can exacerbate the immune response through the activation of macrophages, dendritic cells, and pro-inflammatory helper T lymphocytes (Th1, Th2, and Th17), which begin producing excessive amounts of pro-inflammatory cytokines such as TNF-α, IL-1, IL-6, IL-8, and IL-17 (29). Moreover, increased expression of NF-κB further promotes the production of these cytokines, leading to inflammation and tissue damage, including the intestines (30).

Although previous research has employed different injury models, the structural components involved in inflammatory damage caused by *E. coli* in intestinal cells remain consistent. In this study, animals treated with fish oil showed no progression of hepatic hydropic degeneration. Additionally, the intestinal inflammatory infiltrate in these animals was mild compared to the moderate infiltration observed in untreated animals, suggesting a potential anti-inflammatory effect exerted by fish oil components.

Supplementation with compounds rich in PUFAs appears to positively modulate various aspects of the inflammatory response by inhibiting leukocyte chemokines, adhesion molecules, and inflammatory cytokine production (11). This anti-inflammatory modulation was also demonstrated in a study involving prophylactic treatment with a fish oil nanoemulsion, which significantly reduced inflammation caused by non-typhoidal *Salmonella* infection while preserving intestinal mucosa integrity in murine models (31).

In a clinical study conducted by Zhang et al. (32), sixty-four adult patients with gastrointestinal diseases were randomly assigned to receive parenteral nutrition containing either a fish oil emulsion enriched with ω-3 fatty acids or medium-/long-chain triacylglyceride emulsion for five days post-surgery. The group receiving the ω-3 enriched emulsion showed an increase in leukotriene B5 relative to leukotriene B4 and significant reductions in pro-inflammatory cytokines, including IL-6, TNF-α, and NF-κB. These findings underscore the therapeutic potential of fish oil in treating bacterial infections by promoting mucosal protection and reducing inflammation.

Taken together, our data demonstrated that fish oil exhibited antimicrobial activity against *E. coli* and did not present toxicity to *T. molitor* larvae, HT-29 cells, or mammalian organs. These findings confirm its safety for use in cellular and, by extension, in *in vivo* models. Moreover, fish oil significantly increased the survival rate of larvae infected with EAEC 042 and markedly reduced intestinal colonization in mice infected with the strain. This study is a pioneering effort in exploring this approach and proposes a novel mechanism of action for omega-3-rich fish oil in controlling intestinal infection caused by diarrheagenic *E. coli*. In addition to preserving the integrity of intestinal cells, as evidenced by histological analysis, fish oil showed strong potential as a therapeutic agent for infections caused by this pathogen. Its efficacy highlights its suitability for development into formulations aimed at treating *E. coli*-related illnesses, such as diarrhea.
